# Risk of asthma in patients with primary Sjögren’s syndrome: a retrospective cohort study

**DOI:** 10.1186/s12890-016-0312-3

**Published:** 2016-11-16

**Authors:** Te-Chun Shen, Hsuan-Ju Chen, Chang-Ching Wei, Chia-Hung Chen, Chih-Yen Tu, Te-Chun Hsia, Chuen-Ming Shih, Wu-Huei Hsu, Fung-Chang Sung, Da-Tian Bau

**Affiliations:** 1 Graduate Institute of Clinical Medicine Science, College of Medicine, China Medical University, No. 91 Hsueh-Shih Road, Taichung, 404 Taiwan; 2 Division of Pulmonary and Critical Care Medicine, Department of Internal Medicine, China Medical University Hospital, No. 2 Yu-De Road, Taichung, 404 Taiwan; 3 Management Office for Health Data, China Medical University Hospital, Taichung, 404 Taiwan; 4 Children’s Hospital, China Medical University Hospital, Taichung, 404 Taiwan; 5 Department of Health Services Administration, China Medical University, Taichung, 404 Taiwan; 6 College of Public Health, Kunming Medical University, No. 1168 Chunrongxi Road, Kunming, 650500 YuanNan China; 7 Terry Fox Cancer Research Laboratory, Department of Medical Research, China Medical University Hospital, Taichung, 404 Taiwan; 8 Department of Bioinformatics and Medical Engineering, Asia University, No. 500 Lioufeng Road, Taichung, 41354 Taiwan

**Keywords:** Asthma, Sjögren’s syndrome (SS), Autoimmunity, Retrospective cohort study

## Abstract

**Background:**

Sjögren’s syndrome (SS) has been associated with bronchial hyperresponsiveness and asthma; however, no population-based cohort study has been performed. We evaluated the risk of asthma in patients with primary SS in a nationwide population.

**Methods:**

We conducted a retrospective cohort study using data from the National Health Insurance Research Database in Taiwan. The primary SS group included 4725 adult patients diagnosed between 2000 and 2006. Each patient was frequency-matched with four people without SS by sex, age and year of diagnosis. The occurrence and hazard ratio (HR) of asthma was monitored by the end of 2011.

**Results:**

The overall incidence density of asthma was 1.62-fold higher in the primary SS group than in the non-SS group (9.86 vs. 6.10 per 1000 person-years), with a multivariable Cox proportional hazards model measured adjusted HR of 1.38 [95% confidence interval (CI) = 1.21–1.58]. Stratified analyses by sex, age group, and presence of comorbidity revealed that asthma incidences were all higher in the primary SS group than in the non-SS group, and the relative HRs of asthma associated with primary SS were significant in all subgroups.

**Conclusion:**

Patients with primary SS are associated with an increased risk of developing asthma. We should pay more attention to this group of individuals and provide them with appropriate support.

## Background

Sjögren’s syndrome (SS) is an autoimmune disease characterized by hypofunction of the lacrimal and salivary glands; however, the effects of SS are not limited to the eyes and mouth. SS is also featured by lymphocytic infiltration of exocrine glands. SS can present as an independent disorder itself (primary SS) or as a result of other autoimmune diseases, such as systemic lupus erythematosus (SLE), rheumatoid arthritis (RA), systemic sclerosis, or primary biliary cirrhosis (secondary SS) [1, 2]. Approximately 90% of primary SS cases are females and more prevalent in Caucasian women, with the mean onset age of 40 − 59 [2]. With the incidence of 3.9–5.3 per 100,000 [3, 4], the etiology of SS remains largely unknown. The combination of genetic, environmental, and hormonal factors may trigger the disorder [5]. Patients with SS may develop pulmonary manifestations such as airway disease and interstitial lung disease and follicular bronchiolitis is the most common histologic finding. Patients with significant pulmonary involvement could be at a 4-fold increased risk of death [6]. Therefore, recognition of these conditions is critical in caring for SS patients.

Asthma is a common respiratory disorder characterized by symptoms of wheezing, shortness of breath, chest tightness and cough. The occurrence, intensity, and frequency of these symptoms vary over time, and are associated with variable expiratory airflow, airway wall thickening, and mucus development [7]. Adult- and childhood- onset asthma are quite different; the former is generally non-allergic with severer symptoms and rapid decline in pulmonary function [8]. Pollutants and irritants exposure, upper airway diseases, respiratory infections, female sex hormones, medications, obesity, and stressful life events have been associated with the adult-onset asthma [9].

Previous studies have described SS and the risk of respiratory disorders, including bronchial hyperresponsiveness (BHR) and asthma [10–16]. Fairfax et al. reported that 2 out of 17 (11.8%) patients with SS were also comorbid with asthma [15]. In a large scale case-control study, Kang et al. reported that the prevalence of asthma was greater in the SS cases than in controls (5.1 vs. 3.4%), with an adjusted odd ratio (OR) of 1.54 [95% confidence interval (CI) = 1.22–1.93] [16]. However, the risk of asthma in patients with primary SS compared to the general population is largely unknown.

The National Health Insurance Research Database (NHIRD) of Taiwan is a nationwide database with cohort data including 23 million people. These reliable data have been used for various studies, including several on SS and asthma [17–20]. The hypothesis of this study is that primary SS may increase the likelihood of subsequent diagnosis of asthma. This study was to investigate the risk of asthma in patients with primary SS and compare it with the general population.

## Methods

### Data sources

The National Health Insurance (NHI) is a universal insurance program, which was reformed from 13 insurance systems in 1995. This insurance project has provided comprehensive coverage for >99% of population since 1996. For the present study, we used the Registry of Catastrophic Illnesses Patient Database (RCIPD) and Longitudinal Health Insurance Database 2000 (LHID2000), which are parts of the NHIRD. The RCIPD contains health claims data for people with 30 major diseases, such as cancer, end-stage renal disease, chronic mental illness, and several autoimmune diseases, which are diseases requiring long-term care and are granted with co-pay exemption. The LHID2000 includes longitudinal data on medical claims for 1,000,000 individuals randomly sampled from the 2000 Registry of Beneficiaries. Both RCIPD and LHID2000 contain information on demographic data, clinical visits, prescription details, and diagnostic codes based on the International Classification of Diseases, Ninth revision, Clinical Modification (ICD-9-CM). All personal identifications in the data file were encrypted for privacy protection before being released for research; therefore, written informed consent from the participants involved was unavailable and unnecessary. This study was approved by the Research Ethic Committee of China Medical University and Hospital (CMUH-104-REC2-115).

### Study population

From the data sets of RCIPD and LHID2000, we established two study cohorts for the population-based retrospective cohort study: primary SS cohort and non-SS cohort. Using the data file of RCIPD, adult patients with SS (ICD9-CM code 710.2) newly diagnosed during 2000–2006 were identified as the primary SS cohort. The date that the patient was approved with catastrophic illness certificate was designated as the index date. We excluded patients with secondary SS or a previous diagnosis of asthma (ICD-9-CM code 493). A secondary SS diagnosis was defined as a diagnosis of SS associated with SLE (ICD-9-CM code 710.0), RA (ICD-9-CM code 714), systemic sclerosis (ICD-9-CM code 710.1), or primary biliary cirrhosis (ICD-9-CM code 571.6) [17]. For each patient with primary SS, four insured individuals without SS or asthma were selected from LHID2000 as the non-SS cohort and were frequency-matched based on sex, age (every 5-years span), and year of the index date. The American-European Consensus Criteria was most commonly applied for the diagnosis of SS. According to the criteria, the diagnosis of SS can be met by fulfilling four out of six items, including ocular symptoms, oral symptoms, ocular signs, oral signs, evidence of histopathology, and presence of autoantibodies [21].

### Covariates and outcome

Demographic factors including sex, age (age groups: 20–39, 40–59, and ≥60 years), and medical histories (before the index date) of allergic rhinitis (ICD-9-CM code 477), chronic sinusitis (ICD-9-CM code 473), atopic dermatitis (ICD-9-CM code 691.8), chronic obstructive pulmonary disease (COPD; ICD-9-CM code 496), gastroesophageal reflux disease (GERD; ICD-9-CM codes 530.11 and 530.81), and obesity (ICD-9-CM code 278) were identified as comorbidities.

The primary outcome measure was asthma. Only patients with the diagnosis of asthma (ICD-9-CM code 493) at least twice within 1 year and prescription of treatment for asthma were identified as asthma cases to increase the validity and accuracy. The time of follow-up began with the index date and ended with a new diagnosis of asthma, death, withdrawal from the insurance program, or the end of follow-up on December 31, 2011.

### Statistical analysis

Distributions of sex, age, and comorbidity were compared. The Pearson’s Chi-square test and Student’s *t*-test were used to determine the differences in categorical and continuous variables between the primary SS and non-SS groups. The incidence density of asthma was calculated by dividing the incident number of asthma by the follow-up person-years. We estimated the cumulative incidence of asthma by using the Kaplan-Meier method for the primary SS and non-SS groups and assessed their differences using the log-rank test. We used Cox proportional hazards regression to estimate the hazard ratios (HRs) and 95% CIs of asthma to evaluate the independent effect of primary SS stratified by sex, age, and comorbidity. All statistical analyses were performed using SAS 9.4 (SAS System for Windows, SAS Institute, Cary, NC, USA). Results of comparisons using a two-tailed *p*-value of <0.05 were considered statistically significant.

## Results

This study included 4725 patients with primary SS and 18900 individuals without SS that displayed similar distributions of sex and age (Table 1). The mean age of the primary SS group was 53.1 (SD = 14.0) years, and 87.3% of patients were women. Prevalence rates of allergic rhinitis, chronic sinusitis, atopic dermatitis, COPD, and GERD were all greater in patients with SS than individuals without SS (*p* < 0.001 for all).

**Table 1:** Demographic factors and comorbidities of study participants according to primary Sjögren’s syndrome status

	Non-SS (*N* = 18900)		Primary SS (*N* = 4725)		
Variable	n	%	n	%	*p*-value
Sex					>0.99
Women	16508	87.3	4127	87.3	
Men	2392	12.7	598	12.7	
Age, years					>0.99
20 − 39	3524	18.7	881	18.7	
40 − 59	9532	50.4	2383	50.4	
≥ 60	5844	30.9	1461	30.9	
Means (SD)	52.8	(14.3)	53.1	(14.0)	0.20
Comorbidity					
Allergic rhinitis	1566	8.29	997	21.1	<0.001
Chronic sinusitis	281	1.49	262	5.54	<0.001
Atopic dermatitis	259	1.37	111	2.35	<0.001
COPD	221	1.17	132	2.79	<0.001
GERD	77	0.41	95	2.01	<0.001
Obesity	85	0.45	20	0.42	0.90

*COPD* chronic obstructive pulmonary disease, *GERD* gastroesophageal reflux disease, *SD* standard deviation, *SS* Sjögren’s syndrome

**Fig. 1:**
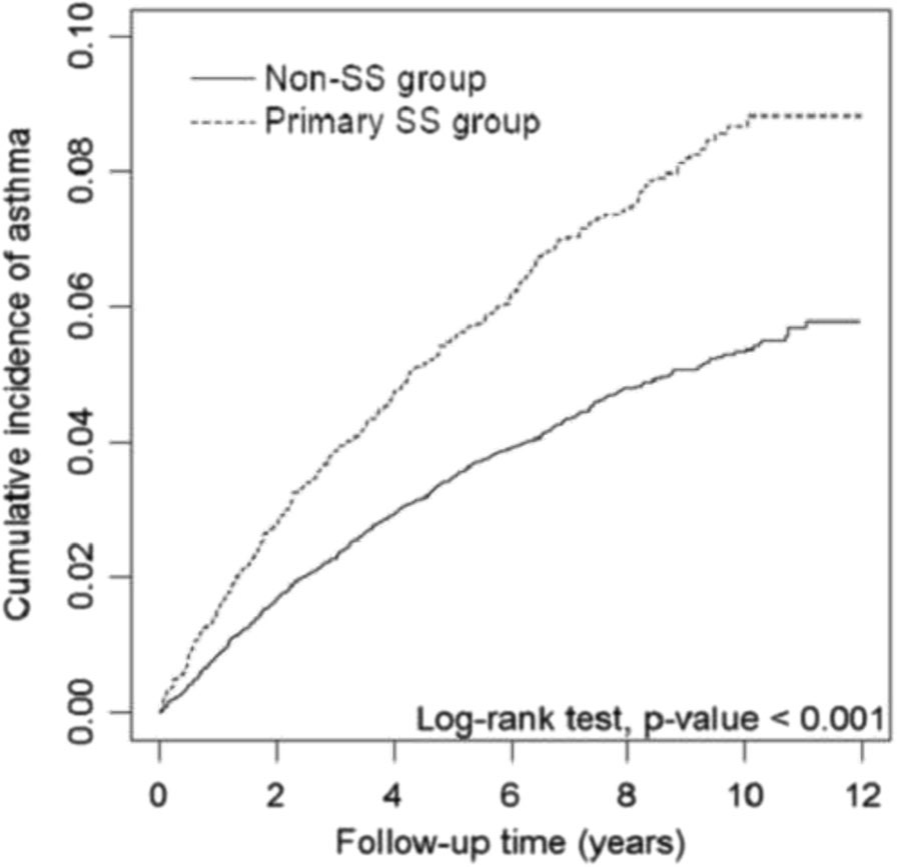
Cumulative incidence of asthma in primary SS group and non-SS group

Figure 1 shows that the cumulative incidence of asthma was 3.2% higher in the primary SS group than in the non-SS group (9.0 vs. 5.8%, *p* < 0.001) after 12-year follow-up. During an average follow-up of 7.31 years, the incidence density of asthma was greater in the primary SS group than in the non-SS group, 9.86 vs. 6.10 per 1000 person-years (Table 2). The multivariable Cox method estimated adjusted HR of asthma was 1.38 (95% CI = 1.21–1.58) for the primary SS group compared with non-SS group after controlling for sex, age, and comorbidities. The adjusted HRs were 1.41 in the 40–59 years age group (95% CI = 1.16–1.70) and 2.65 in the ≥60 years age group (95% CI = 2.19–3.22), compared with those aged less than 40 years. Compared with individuals free of COPD, those with COPD had an adjusted HR of 2.52 (95% CI = 1.91–3.33) for developing asthma. On the other hand, adjusted HRs were 1.76 (95% CI = 1.51–2.06) for individuals with allergic rhinitis, 1.71 (95% CI = 1.31–2.22) for individuals with chronic sinusitis, and 2.05 (95% CI = 1.10–3.82) for individuals with obesity.

**Table 2:** Cox model measure hazard ratios and 95% confidence intervals of asthma associated with primary Sjögren’s syndrome and covariates

Variables	Events	IR	HR (95% CI)	
			Univariate	Multivariate^a^
Primary SS				
No	844	6.10	1.00	1.00
Yes	337	9.86	1.62 (1.42 − 1.83)***	1.38 (1.21 − 1.58)***
Sex				
Women	1007	6.62	1.00	1.00
Men	174	8.45	1.26 (1.07 − 1.48)**	0.90 (0.76 − 1.06)
Age, years				
20 − 39	134	3.95	1.00	1.00
40 − 59	504	5.61	1.41 (1.17 − 1.71)***	1.41 (1.16 − 1.70)***
≥ 60	543	11.1	2.74 (2.27 − 3.31)***	2.65 (2.19 − 3.22)**
Comorbidity				
Allergic rhinitis				
No	952	6.13	1.00	1.00
Yes	229	13.1	2.08 (1.80 − 2.41)***	1.76 (1.51 − 2.06)***
Chronic sinusitis				
No	1116	6.61	1.00	1.00
Yes	65	17.5	2.58 (2.01 − 3.32)***	1.71 (1.31 − 2.22)***
Atopic dermatitis				
No	1153	6.77	1.00	1.00
Yes	28	11.8	1.67 (1.15 − 2.43)**	1.39 (0.95 − 2.02)
COPD				
No	1123	6.58	1.00	1.00
Yes	58	29.8	4.30 (3.30 − 5.60)***	2.52 (1.91 − 3.33)***
GERD				
No	1167	6.80	1.00	1.00
Yes	14	15.2	2.03 (1.20 − 3.41)**	1.28 (0.75 − 2.17)
Obesity				
No	1171	6.81	1.00	1.00
Yes	10	14.1	2.02 (1.09 − 3.77)*	2.05 (1.10 − 3.82)*

*CI* confidence interval, *COPD* chronic obstructive pulmonary disease, *GERD* gastroesophageal reflux disease, *HR* hazard ratio, *IR* incidence density rate per 1000 person-years, *SS* Sjögren’s syndrome

^a^Model was adjusted for sex, age and comorbidity in Cox proportional hazards

regression

*p <0.05, **p <0.01, ***p <0.001

Table 3 shows incident asthma for both study cohorts by sex, age and comorbidity status. The primary SS group to the non-SS group adjusted HR was significant for women (1.39, 95% CI = 1.21–1.60). Age stratification demonstrated that incidence of asthma increased with age in both cohorts. The primary SS to non-SS group hazard ratio was greater for younger individuals. For individuals without comorbidity, those in the primary SS group had an adjusted HR of 1.47 (95% CI = 1.25–1.72) compared with those in the non-SS group.

**Table 3:** Incidence density rates and hazard ratios of asthma according to primary Sjögren’s syndrome status stratified by sex, age, and comorbidity

	No-SS group			Primary SS group			Compared to the non-SS group	
							HR (95% CI)	
Variables	Events	Person-years	IR	Events	Person-years	IR	Relative	Adjusted^b^
Sex								
Women	720	121936	5.90	287	30090	9.54	1.61 (1.41 − 1.85)***	1.39 (1.21 − 1.60)***
Men	124	16497	7.52	50	4104	12.2	1.63 (1.17 − 2.27)**	1.31 (0.92 − 1.86)
Age, years								
20 − 39	87	27166	3.20	47	6797	6.91	2.17 (1.52 − 3.09)***	1.80 (1.24 − 2.60)**
40 − 59	351	72063	4.87	153	17771	8.61	1.77 (1.46 − 2.13)***	1.54 (1.26 − 1.87)***
≥ 60	406	39204	10.4	137	9626	14.2	1.37 (1.13 − 1.67)**	1.16 (0.95 − 1.42)
Comorbidity status^a^								
No	658	123800	5.32	196	25269	7.76	1.47 (1.25 − 1.72)***	1.47 (1.25 − 1.72)***
Yes	186	14633	12.7	141	8925	15.8	1.25 (1.01 − 1.56)*	1.24 (0.99 − 1.54)

*CI* confidence interval, *HR* hazard ratio, *IR* incidence density rate per 1000 person-years, *SS* Sjögren’s syndrome

^a^Individuals with any one of allergic rhinitis, chronic sinusitis, atopic dermatitis, chronic obstructive pulmonary disease, gastroesophageal reflux disease, and obesity were classified as the comorbidity group

^b^Model was adjusted for sex, age and comorbidity in Cox proportional hazards regression

*p <0.05, **p <0.01, ***p <0.001

## Discussion

To the best of our knowledge, this is the first nationwide population-based retrospective cohort study to investigate the risk of asthma in patients with primary SS, comparing with general population. Results revealed that patients with primary SS had an increased risk of asthma compared to those without SS. Stratified analyses by sex, age group, and presence of comorbidity also showed that the incidence rates of asthma were consistently higher in primary SS patients than in the comparison group, and the relative HRs of asthma associated with primary SS were significant for all subgroups. However, there were reduced significance levels in calculating adjusted HRs for some subgroups including males, those aged >60 years, and those with any comorbidity. The possible explanation for this may be that the case numbers were reduced in these subgroups, which in turn influenced the statistical power.

Asthma is a heterogeneous disease associated with different underlying disease processes and pathophysiologies. Recently, cluster analysis have classified asthma phenotypes by clusters of demographic, clinical, or pathophysiological characteristics [7]. A large number of studies have documented that adults with asthma may not associate with allergies, and the cellular profile of their airways may be neutrophilic, eosinophilic, or other types. These patients are classified as having non-allergic or adult-onset (late-onset) asthma, and they often respond less well to inhaled corticosteroid treatment [7]. Primary SS patients with BHR have characteristics similar to those with non-allergic or adult-onset asthma. Papiris et al. have reported that CD4 positive T-lymphocytes in the bronchial mucosa are increased outside of the bronchial glands in patients with SS, supporting the fact that SS involves extra-glandular tissues in the airways [22]. Amin et al. found that number of neutrophils, mast cells and T lymphocytes in the airways were higher in patients with primary SS than in healthy controls, while the number of eosinophils was similar in SS patients and controls [23]. Stalenheim et al. reported that inhaled budesonide was less effective for alleviating BHR in patients with SS [24]. In addition, Amin et al. have observed a lowered degree of epithelial integrity, but thicker tenascin and laminin layers in primary SS patients than in controls [23]. They concluded that structural changes in the airways relating to BHR were similar in SS patients and asthma patients [23]. Therefore, it is reasonable to include primary SS patients with BHR and related respiratory symptoms into a certain phenotype of asthma, which met the clinical diagnostic criteria.

Tobacco smoking has been considered as a potential confounding factor in the present study because the NHIRD does not contain data on personal smoking habits. One study has reported a weak association between cigarette smoking and the development of primary SS [25], while most studies reported no significant difference in smoking between primary SS patients and controls [26–28]. The case-control study on cardiovascular diseases even found significantly fewer smokers in the SS patient groups [29, 30]. Therefore, patients with SS may not have a significantly higher smoking rate than those without SS [18].

In the present study, we were able to observe a “real world” scenario using the claims data, in which primary SS, asthma, and comorbidities were directly diagnosed during medical consultations. In terms of the disease definition and patient registry, SS is categorized as a “catastrophic illness” and patients diagnosed with SS are entitled to receive the “catastrophic illness certification” issued by the government. Experts specializing in the disease make critical evaluation of claims data, including serological, and/or pathological reports with reference to the American-European Consensus Criteria [21]. The diagnosis of asthma depends on a target history and requires comprehensive evidence of variable expiratory airflow limitations. We typically follow the Global Initiative for Asthma (GINA) guideline to arrive at an asthma diagnosis [7]. The insurance authority has established a committee to evaluate the claims data to prevent errors and violations. In addition, we only selected those diagnoses (asthma and comorbidities) that appeared at least twice within 1 year to increase the validity and accuracy of the diagnosis.

The universal coverage of the NHI project reduces barriers to health care access for all insured population, regardless of socioeconomic status [31]. In an earlier case-control study evaluating comorbidities for patients with primary SS, Kang et al. reported an odds ratio of 1.54 for asthma risk in primary SS patients, which is similar to our findings [16]. We suggest further evaluation of the differences in medications prescribed for asthma patients with primary SS compared to those without SS to better understand the current and common medical treatments for this population. In addition, future studies should evaluate the treatment outcomes such as acute exacerbations, admissions, intensive care, or mortality due to asthma in patients with primary SS.

The strength of this study comes from using the longitudinal population-based data to evaluate the asthma risk in primary SS patients. It is generally costly to conduct a population-based prospective cohort study. A retrospective cohort study based on insurance or registry data is an economically suitable alternative to evaluate this relationship, full compliance with the requirements of a follow-up study. However, several limitations of the study must be considered when interpreting the present findings. First, the NHIRD does not provide detailed information on potential confounding factors for this study, such as environmental factors, personal habits, diet preference, and family history. Since the crucial information is missing, it is difficult to judge whether we have used the most suitable comparison individuals. In addition, relevant clinical variables, such as image reports, histopathology results, serum laboratory data, pulmonary function tests, and symptom frequency in patients were also unavailable. We were unable to evaluate whether there is dose-response relationship between asthmatic risk and symptom frequency in SS patients.

## Conclusion

Patients with primary SS are associated with a higher risk of developing asthma than those without SS. The association between primary SS and allergic or non-allergic asthma needs further investigation. In any case, we should pay more attention to patients with primary SS and provide them with appropriate support.
